# Association of Plasma Irisin Levels with Circulating Endothelial Microparticles (EMPs) and Endothelial Progenitor Cells (EPCs) in Children Born Prematurely

**DOI:** 10.3390/metabo13010120

**Published:** 2023-01-13

**Authors:** Panagiota Markopoulou, Arsinoi Koutroumpa, Aimilia Mantzou, Alexandra Margeli, Eleni Papanikolaou, Tania Siahanidou

**Affiliations:** 1 Neonatal Unit, First Department of Pediatrics, School of Medicine, National and Kapodistrian University of Athens, 11527 Athens, Greece; 2 Second Neonatal Intensive Care Unit, “Agia Sofia” Children’s Hospital, 11527 Athens, Greece; 3 Unit of Clinical and Translational Research in Endocrinology, First Department of Pediatrics, School of Medicine, National and Kapodistrian University of Athens, 11527 Athens, Greece; 4 Department of Clinical Biochemistry, “Agia Sofia” Children’s Hospital, 11527 Athens, Greece; 5 Laboratory of Biology, School of Medicine, National and Kapodistrian University of Athens, 11527 Athens, Greece

**Keywords:** prematurity, endothelial dysfunction, endothelial microparticles, endothelial progenitor cells, irisin, children

## Abstract

Prematurity has been linked with endothelial dysfunction in later life. The purpose of this study was to evaluate the association between plasma irisin, an adipomyokine reported to protect the functional integrity of vascular endothelium, and circulating endothelial microparticles (EMPs) and endothelial progenitor cells (EPCs), consisting early biomarkers of endothelial dysfunction, in preterm-born children. We studied 131 prepubertal children; 61 preterm and 70 born at term (controls). Plasma irisin was determined by ELISA. Circulating CD62E(+), CD144(+) and CD31(+)/CD42b(-) EMPs, and CD34(+)/VEGFR-2(+)/CD45(-) and CD34(+)/VEGFR-2(+)/CD45dim EPCs, were determined by flow cytometry. Body mass index, waist-to-hip ratio, neck circumference, systolic and diastolic blood pressure, and biochemical parameters (glucose, lipids, insulin, HOMA-IR) were also evaluated. Plasma irisin was significantly lower (*p* = 0.001), whereas circulating EMPs and EPCs were higher, in children born prematurely compared to controls. Irisin was recognized as independent predictor for CD144(+) and CD31(+)/CD42b(-) EMPs, CD34(+)/VEGFR-2(+)/CD45(-) and CD34(+)/VEGFR-2(+)/CD45dim EPCs in the total study population, and for CD31(+)/CD42b(-) EMPs in the preterm group. In conclusion, plasma irisin correlates independently with circulating EMP and EPC subpopulations in prepubertal children and in preterm-born ones. Further studies in children will potentially elucidate the link between irisin and the primary stages of prematurity-related endothelial dysfunction.

## 1. Introduction

A growing body of evidence has revealed the negative effect of preterm birth on cardiovascular health and endothelial function in later life [1,2,3,4,5]. Potential pathways linking prematurity with increased risk of cardiovascular disease and endothelial dysfunction have been described; increased production of reactive oxygen species levels and/or decreased intracellular antioxidant capacity [3], immature inflammatory process [6], increased circulating endothelial microparticles (EMPs) and endothelial progenitor cells (EPCs) [7,8] or dysfunction of EPC subpopulations, such as endothelial colony-forming cells (ECFCs) [9] in preterm-born individuals are possible pathophysiological mechanisms. 

Circulating EMPs are small vesicular structures, <1.0 μm in size, released from endothelial cells in response to cellular inflammatory activation, apoptosis or injury [10]. EMPs have been proposed as early biomarkers of endothelial activation and damage [10]; increased numbers of EMPs are indicative of endothelial dysfunction [10,11,12]. Previous studies have demonstrated that circulating EMPs are higher in children born prematurely [7]. 

Circulating EPCs are considered as precursors of mature endothelial cells; they originate from bone marrow and they can be isolated from the peripheral and umbilical cord blood [13]. They are important for vascular homeostasis and repair, for remodeling of existing blood vessels and growth of new vessels, as well as for tissue regeneration after ischemia and for the neovascularization physiological process [13,14]. In preterm infants, circulating EPCs have no difference or they are increased in comparison with full-term individuals [15], while in prepubertal children born prematurely, EPCs are higher compared to controls [8]. 

Irisin is a newly discovered adipomyokine produced principally in the skeletal muscle after acute exercise [16], and to a lesser degree in adipose tissue [17]. It was first identified in muscle cells of the transgenic mice overexpressing the Ppargc1a gene, encoding the transcription cofactor peroxisome proliferator-activated receptor-γ co-activator 1α (PGC-1α), which is involved in energy metabolism regulation [16]. PGC-1α has been reported to enhance the expression of the transmembrane fibronectin type III domain-containing protein 5 (FNDC5), and after several modifications including glycosylation of FNDC5, the 112-amino acid peptide irisin is released [16,18]. After secretion from muscle, irisin stimulates the expression of uncoupling protein 1 (UCP1) in adipocytes, leading to “browning” of white adipose tissue [16,19]; it also increases energy expenditure and thermogenesis, and improves insulin sensitivity [16]. In adipose tissue, irisin inhibits lipid synthesis, stimulates lipolysis, and reduces the differentiation of preadipocytes, thus resulting in decreased fat mass [18,20]. Furthermore, irisin significantly increases the uptake of glucose and fatty acids in muscle cells, while the secreted irisin from hepatocytes may reduce gluconeogenesis by acting in an autocrine and/or paracrine manner [18,20].

Previous studies have investigated irisin and its possible associations with cardiometabolic parameters among both adults and children, with conflicting results; obese patients, patients with diabetes mellitus type 2 and/or patients with metabolic syndrome have been described to have both higher [21,22] and lower irisin levels compared to controls [23,24,25,26,27], while irisin levels have displayed positive, as well as negative associations with adiposity parameters, lipid profile and insulin resistance [21,27,28,29]. The possible effect of irisin on cardiometabolic parameters has not been fully elucidated. Evidence indicates the potential effect of irisin on endothelial function; irisin was found to increase the number of EPCs in peripheral blood and also improve the function of bone marrow-derived EPCs in mice with diabetes mellitus [30]. Furthermore, irisin may improve EPC concentrations, migratory and adhesive functions [31], and may also strengthen the endothelial junctions and the function of endothelial barrier by binding to integrin αVβ5 receptor in endothelial cells [32]. In obese children, irisin has been found to display inverse correlations with inflammatory markers of endothelial activation [24]. 

Regarding the effect of preterm birth on irisin levels, previous studies have shown lower circulating irisin levels in children born prematurely compared to full-term born peers [33]; furthermore, lower irisin levels have been observed in preterm-born infants compared to infants born at term [34,35]. However, the effect of irisin on cardiovascular risk and endothelial dysfunction of preterm-born individuals is still under investigation. To the best of our knowledge, there are no published studies that have examined the possible associations between irisin levels and circulating EMPs or EPCs among the population of preterm-born children in the context of endothelial dysfunction related to prematurity.

The purpose of this study was to investigate the circulating irisin levels and their relation to circulating endothelial microparticles (EMPs) and endothelial progenitor cells (EPCs) in a population of children born prematurely compared to children born at term.

## 2. Materials and Methods

### 2.1. Study Design and Population

In this cross-sectional observational study, 131 prepubertal children (mean age 10.7 ± 1.8 years), born between 2007 and 2011, were enrolled. Of them, 61 children (24 males and 37 females) were born prematurely (<37 weeks of gestational age). Among preterm-born children, 50 had birth weight appropriate for gestational age (AGA), 10 were small for gestational age (SGA) and one was large for gestational age (LGA); AGA was defined as birth weight between 10th and 90th percentiles for gestational age and gender, whereas SGA as birth weight below the 10th percentile, and LGA as birth weight above the 90th percentile for gestational age and gender [36]. The control group consisted of 70 healthy children (36 males and 34 females) born at term (37–42 weeks of gestational age); of them, 58 were AGA, 8 were SGA and 4 were LGA. 

The study population was derived and extended from a previous study investigating circulating EMPs and EPCs [7,8]; both studies evaluated the associations between circulating EMPs and EPCs with cardiovascular risk factors and several endothelial parameters in prepubertal preterm-born children versus children of similar age who were born full-term. All participants were hospitalized as neonates at “Agia Sofia” Children’s Hospital, Athens, Greece, and followed-up regularly in the follow-up clinic. 

In the study population, medical and family history was obtained, whereas perinatal and neonatal data and morbidity, and pregnancy complications were extracted from hospital medical records. Maternal gestational diabetes was defined as glucose intolerance, of any degree, with onset or first recognition during pregnancy [37]. Concerning neonatal morbidity, bronchopulmonary dysplasia (BPD) was defined as supplemental oxygen requirements at 36 weeks post-menstrual age [38]. Since exercise could account for differences in irisin levels [39], physical activity of the participants was also assessed by use of Physical Activity Rating (PA-R) questionnaire, providing a grade from 0 to 7 [40]. 

Children with a family history of cardiovascular disease, or with congenital anomalies, or active disease or obesity (body mass index (BMI) ≥ 95th percentile for age) were excluded. The study protocol was approved by the Research and Ethics committee of “Agia Sofia” Children’s Hospital and informed written consent was obtained from parents and children.

### 2.2. Sample Size Calculation

A statistical power analysis was performed for sample size estimation based on data from previous studies that investigated potential differences in circulating irisin levels between preterm-born and full-term-born individuals [33,34], as well as previous studies that investigated possible correlations of irisin levels with EMP and EPC subpopulations [24,31]. Regarding previous studies that examined irisin levels in preterm-born individuals compared to individuals born at term, the effect size (ES) was medium using Cohen’s (1988) criteria; ES = 0.52 [33] and ES = 0.55 [34]. With an alpha = 0.05 and power = 0.80, the projected sample size needed with this effect size (GPower 3.1.9.2 software) was found to be at least N = 53 for each of the groups of children studied. 

Since our study was the first to examine possible associations of irisin levels with circulating EMPs and EPCs in prepubertal children born prematurely, the a priori statistical power analysis for these associations was based on studies conducted on other groups of children (e.g., obese and normal-weight children) and focused on the potential role of irisin on their endothelial function [24,31]. The correlation coefficient effect size (ES) in the studies used was medium to large for EMPs and EPCs subpopulations studied using Cohen’s (1988) criteria; correlation coefficient ES = 0.62 for EMPs subpopulations [24] and correlation coefficient ES = 0.52 for EPCs subpopulations [31]. With an alpha = 0.05 and power = 0.80, the projected total sample size needed with this effect size (GPower 3.1.9.2 software) was found to be at least N = 15 for significant correlations between irisin levels and EMPs, and at least N = 24 for significant correlations between irisin levels and EPCs. 

Thus, our sample size was more than adequate to detect possible differences in irisin levels between prepubertal children born prematurely and those born at term or the associations of irisin with EMP and EPC subpopulations.

### 2.3. Clinical Assessment

All the participants attended the outpatient clinic at 7.30–9.30 a.m. after a 12-h overnight fast. Standing height was measured to the nearest 0.1 cm on bare foot using a Harpenden stadiometer (London, UK). Body weight was assessed to the nearest 0.1 kg using a scale (Seca 712, UK) with subjects wearing light clothing [7,8]. Waist and hip circumference were measured to the nearest 0.1 cm over an unclothed abdomen and at minimal respiration [7,8,41]. The waist/hip ratio (WHR) and the BMI, as weight (kg)/[height (m^2^)] ratio, were calculated. Neck circumference was measured at the level of the most prominent portion, the thyroid cartilage, with the neck in a horizontal plane [42]. Each anthropometric measurement was done twice and the mean was used for analyses. Furthermore, measurements of systolic (SBP) and diastolic blood pressure (DBP) were performed three times in all participants, using an automatic oscillometric blood pressure monitor and with an appropriate-sized cuff [43], after a 10-min rest in the sitting position; the 2nd and 3rd measurements were averaged for analyses [43,44].

### 2.4. Blood Biochemistry and Plasma Irisin Levels

Fasting glucose, insulin, total cholesterol, high-density lipoprotein cholesterol (HDL-C), low-density lipoprotein cholesterol (LDL-C), and triglycerides serum levels were measured in all children studied. The homeostasis model assessment of insulin resistance (HOMA-IR) index was calculated (insulin (μU/mL) × glucose (mmol/L)/22.5) [45].

Irisin concentrations were determined in plasma using the irisin recombinant ELISA kit (Phoenix Pharmaceuticals, Inc., Burlingame, CA, USA). The intra-assay and inter-assay coefficients of variation (CVs) were ≤10% and ≤15%, respectively; sensitivity was 2.42 ng/mL and specificity was 100% for human irisin (1–112) and 9% for FNDC5 isoform 4. 

### 2.5. Flow Cytometric Analysis of Circulating Endothelial Microparticles (EMPs) and Endothelial Progenitor Cells (EPCs)

Blood samples were collected in 3.2% sodium citrate tubes in an atraumatic fashion; the first 5 mL of collected blood was used for routine blood tests, including glucose, insulin and lipid profile, in order to avoid contamination with EMPs due to vascular injury. Circulating EMPs were phenotyped and quantified by flow cytometry in platelet-poor plasma (PPP), after a two-step centrifugation protocol (860× *g* for 20 min at 4 °C, followed by 1500× *g* for 5 min at 20 °C), within one to two hours after collection, as previously described [7,46]; 50 μL of PPP was incubated with fluorochrome labeled antibodies specific for CD31 [CD31-FITC (ImmunoTools)], CD62E [CD62E-PE (Santa Cruz Biotechnology)], CD144 [CD144-PerCP (Santa Cruz Biotechnology)], and CD42b [CD42b-APC (ImmunoTools)], for 20 min at 4 °C in the dark. Afterwards, samples were diluted with phosphate buffered saline (PBS) and then they were analyzed by flow cytometry. Three EMPs subpopulations were identified as CD62E(+), CD144(+), or CD31(+)/CD42b(-) events; values are reported as the percentage of each EMP subpopulation among the total microparticle population. CD62E(+) and CD144(+) EMP subpopulations are microparticles expressing CD62E and CD144 surface markers respectively, whereas CD31(+)/CD42b(-) EMP subpopulation consists of microparticles expressing CD31, but not the CD42b surface marker.

For detection and quantification of EPCs, peripheral-blood mononuclear cells (PBMCs) were isolated and immediately stored at –80 °C in freezing medium containing 10% (*v*/*v*) DMSO [8]. EPCs were phenotyped and measured by flow cytometry, as previously described [8]; for quantification of CD34(+)/VEGFR-2(+)/CD45(-) and CD34(+)/VEGFR-2(+)/CD45dim endothelial progenitor cells, initially we gated CD45(-) and CD45dim PBMCs. Afterwards, the dual expression of CD34 and VEGFR-2 was determined within the CD45(-) and CD45dim populations.

Both circulating EMPs and EPCs were measured using a fluorescence activated cell sorter (FACS) Calibur flow cytometer (BD FACSCalibur^TM^, BD Biosciences, San Jose, CA, USA). For data analysis, the Flowing Software version 2.5.1. was used. All measurements were performed blinded to gestational age and to clinical and laboratory characteristics of study participants.

### 2.6. Statistical Analyses

Statistical analyses were conducted using SPSS version 23.0 (SPSS Inc., Chicago, IL, USA). Normality of distribution was examined using the Kolmogorov–Smirnov and Shapiro–Wilk tests. Results are expressed as mean ± SD for parameters with normal distribution, or as median (25th–75th percentile) for parameters not normally distributed. Groups were compared using t-test or Mann–Whitney U test, as appropriate, for continuous variables and chi-square test for categorical variables. For linear correlations between variables of interest, Pearson’s or Spearman’s correlation coefficient was used for parameters with normal or skewed distribution, respectively; multiple stepwise regression analysis was further applied to examine independent associations of EMPs and EPCs with irisin levels. Due to skewed distribution, log-transformation of EMPs and EPCs subpopulations was performed before analyses. Statistical significance level was set at *p*-value ≤ 0.05.

## 3. Results

Table 1 presents the baseline clinical, neonatal and perinatal characteristics, as well as the biochemical profile of study participants. No differences in age, sex and physical activity were observed between preterm-born children and controls. Mean ± SD gestational age and birth weight of preterm-born children were 31.7 ± 3.2 weeks and 1619 ± 540 g, respectively. Children born prematurely displayed higher waist circumference (*p* = 0.02), WHR (*p* = 0.05) and neck circumference (*p* = 0.05) in comparison with controls. Furthermore, children born prematurely presented with significantly higher levels of both SBP (*p* = 0.01) and DBP (*p* = 0.04) compared to controls. No significant differences were found in glucose, insulin, HOMA-IR, or lipid levels between children born prematurely and controls (Table 1).

**Figure 1:**
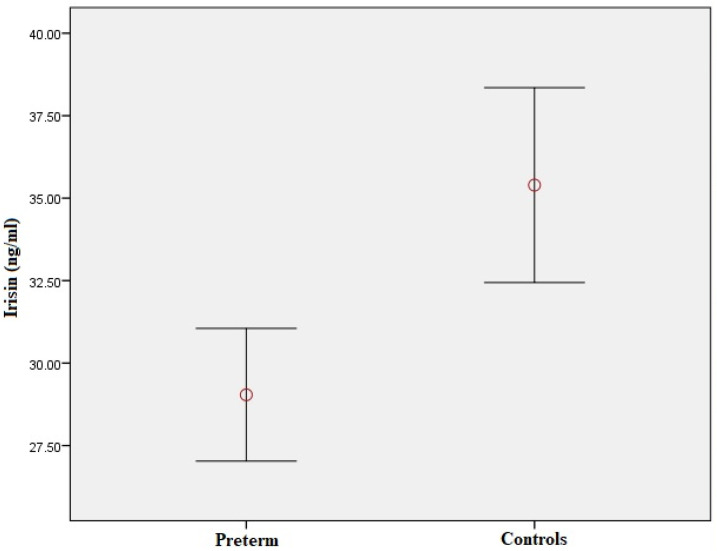
Plasma irisin levels in preterm-born children compared to controls. Red circle represents the mean value of plasma irisin levels in children born prematurely and controls respectively; error bars represent standard error of mean.

**Figure 2:**
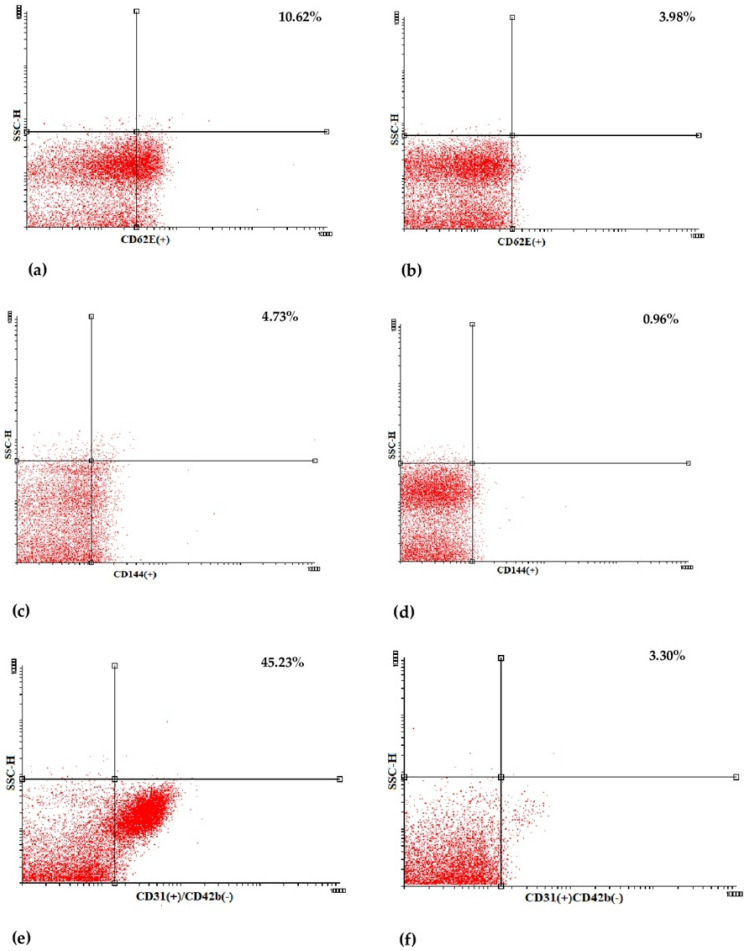
Representative dot plots of flow cytometric determination of CD62E(+) (**a**,**b**), CD144(+) (**c**,**d**) and CD31(+)/CD42b(-) (**e**,**f**) endothelial microparticles (EMPs) in a preterm-born child (**left**) and in a full-term-born child (**right**). SSC-H: side scatter height.

**Figure 3:**
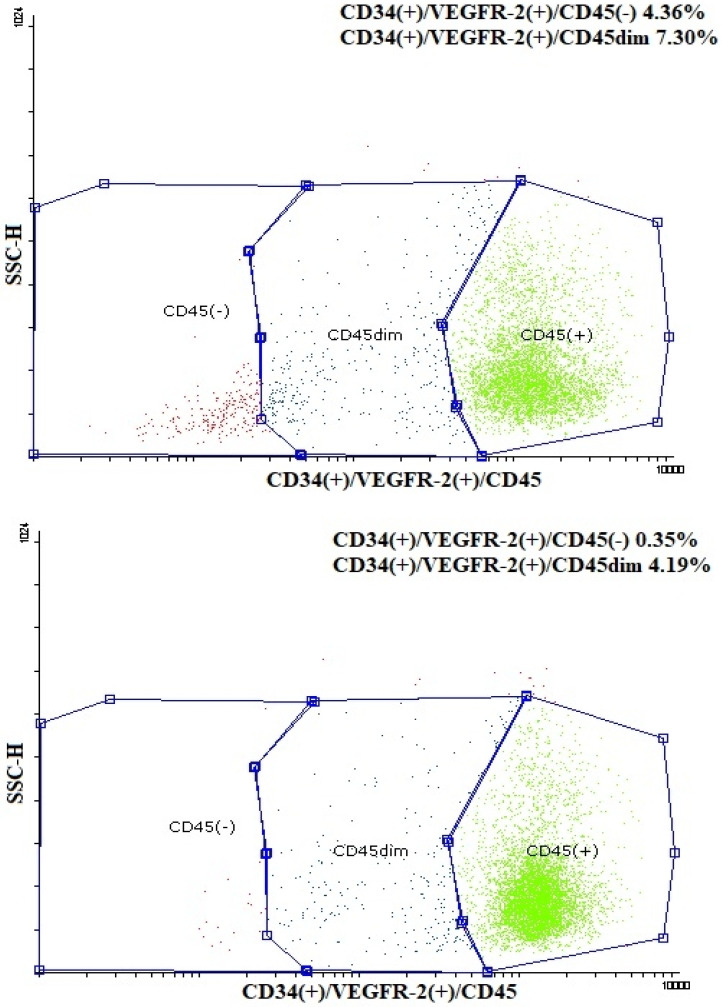
Representative dot plots of flow cytometric determination of CD34(+)/VEGFR-2(+)/CD45(-) and CD34(+)/VEGFR-2(+)/CD45dim endothelial progenitor cells (EPCs) in a child born prematurely (**up**) and in a child born at term (**down**). SSC-H: side scatter height.

Plasma irisin levels were significantly lower in preterm-born children compared to controls (29.0 ± 7.9 vs. 35.4 ± 12.4 ng/mL, *p* = 0.001) (Figure 1); whereas, regarding circulating EMPs, CD62E(+) [8.71 (6.52–12.36)% vs. 7.41 (4.67–9.56)%, *p* = 0.01] (Figure 2a,b and Figure S1a), CD144(+) [1.69 (0.65–4.47)% vs. 0.91 (0.42–1.56)%, *p* < 0.001] (Figure 2c,d and Figure S1b) and CD31(+)/CD42b(-) [24.35 (8.87–53.65)% vs. 7.41 (3.29–14.99)%, *p* < 0.001] (Figure 2e,f and Figure S1c) EMPs were significantly higher in preterm-born children compared to controls. Furthermore, in children born prematurely, circulating CD34(+)/VEGFR-2(+)/CD45(-) and CD34(+)/VEGFR-2(+)/CD45dim EPCs were significantly higher in comparison with controls [0.59 (0.15–2.60)% vs. 0.15 (0.06–0.47)%, *p* < 0.001 and 8.94 (4.41–23.17)% vs. 3.08 (1.05–7.97)%, *p* < 0.001, respectively] (Figure 3 and Figure S2). No differences were found between preterm-born males and preterm-born females regarding irisin levels, circulating EMP or EPC subpopulations studied.

### 3.1. Correlation of Irisin with Clinical, Perinatal and Biochemical Variables 

In the total study population, irisin levels were negatively correlated with the age of participants (r_s_ = −0.19, *p* = 0.03), while they were positively correlated with gestational age (r_s_ = 0.20, *p* = 0.02) and birth weight (r_s_ = 0.23, *p* = 0.01). Furthermore, irisin correlated positively with WHR in the total study population (r_s_ = 0.19, *p* = 0.03) and in the preterm group (r_s_ = 0.35, *p* = 0.01). No significant correlations were found between irisin and glucose, insulin, or lipid levels, HOMA-IR, or physical activity score in preterm-born children and controls.

### 3.2. Correlation of Irisin with Circulating EMPs and EPCs

In the total study population, irisin levels were negatively correlated with CD62E(+) (Figure S3a), CD144(+) (Figure S3b) and CD31(+)/CD42b(-) (Figure S3c) EMPs (r_s_ = −0.19, *p* = 0.03; r_s_ = −0.24, *p* = 0.01; r_s_ = −0,31, *p* < 0.001, respectively). In the preterm-born group, circulating irisin levels were also negatively correlated with CD62E(+) (Figure S4a) and CD31(+)/CD42b(-) (Figure S4b) EMPs (r_s_ = −0.27, *p* = 0.03 and r_s_ = −0.36, *p* = 0.004, respectively). In the control group, no significant correlations between circulating irisin levels and EMPs emerged.

Regarding circulating EPCs, irisin levels correlated negatively with CD34(+)/VEGFR-2(+)/CD45(-) (Figure S3d) and CD34(+)/VEGFR-2(+)/CD45dim (Figure S3e) EPCs (r_s_ = −0.23, *p* = 0.01 and r_s_ = −0.34, *p* < 0.001, respectively) in the entire study population. In the preterm-born group, circulating irisin levels were also negatively correlated with CD34(+)/VEGFR-2(+)/CD45dim EPCs (r_s_ = −0.26, *p* = 0.04) (Figure S4c). In the control group, circulating irisin levels were negatively correlated with CD34(+)/VEGFR-2(+)/CD45(-) and CD34(+)/VEGFR-2(+)/CD45dim EPCs (r_s_ = −0.25, *p* = 0.04 and r_s_ = −0.29, *p* = 0.01, respectively). 

Multiple regression analyses were further run, with each EMP and EPC subpopulation as a dependent variable, in order to evaluate independent associations between circulating EMPs and EPCs and plasma irisin, anthropometric and metabolic parameters (Table 2). After adjusting for age, sex, born SGA, BMI, WHR, HOMA-IR and lipid levels, plasma irisin was recognized as an independent predictor for CD144(+) (Table 2) and CD31(+)/CD42b(-) EMPs (Table 2B), as well as for CD34(+)/VEGFR-2(+)/CD45(-) (Table 2) and CD34(+)/VEGFR-2(+)/CD45dim EPCs (Table 2) in the total study population. Furthermore, in the total study population, WHR correlated significantly with CD31(+)/CD42b(-) EMPs (Table 2), while age and triglyceride levels correlated significantly with CD34(+)/VEGFR-2(+)/CD45(-) (Table 2) and CD34(+)/VEGFR-2(+)/CD45dim EPCs (Table 2). In the preterm-born group, plasma irisin levels and SGA were independently associated with CD31(+)/CD42b(-) EMPs (Table 2).

## 4. Discussion

In this study, plasma irisin levels were found to be significantly lower in prepubertal children born prematurely compared to children born at term. The preterm-born group also presented an adverse cardiometabolic phenotype, with higher waist and neck circumference, waist-to-hip ratio, and levels of both SBP and DBP in comparison with controls. Furthermore, circulating CD62E(+), CD144(+) and CD31(+)/CD42b(-) EMPs and CD34(+)/VEGFR-2(+)/CD45(-) and CD34(+)/VEGFR-2(+)/CD45dim EPCs were significantly higher in the preterm-born group compared to children born at term, which indicates that preterm birth is possibly linked with endothelial activation and damage that is already evident in childhood. Circulating EMPs and EPCs correlated negatively with plasma irisin levels. 

To the best of our knowledge, this is the first study to examine the associations of plasma irisin concentrations with circulating EMPs and EPCs in preterm-born individuals. In general, it is widely accepted that endothelial dysfunction is a significant regulator in the pathogenesis and development of cardiovascular disease and/or metabolic syndrome [47,48]. Limited studies have investigated the relationship between irisin and endothelial dysfunction parameters [30,32]; irisin may have a protective role against endothelial barrier dysfunction by strengthening the endothelial junctions via binding to integrin αVβ5 receptor and improving mitochondrial function in endothelial cells [32]. Furthermore, it was shown previously that irisin improves the proliferative and migratory capacities of EPCs in peripheral blood and enhances endothelial repairing via the PI3K/Akt/eNOS pathway in an animal model of diabetes mellitus [30]. Moreover, in obese adults, an increase in circulating levels of irisin following an exercise training and dietary restriction program was associated with improvement in EPCs concentrations, migratory and adhesive functions [31]. In pediatric populations, irisin was found to display inverse correlations with inflammatory markers of endothelial activation, such as E-selectin and ICAM-1, in obese children [24]. However, the effect of irisin on circulating EMPs has not been further studied so far.

In this study, plasma irisin levels were inversely correlated with EMPs subpopulations similarly to previous studies in high-risk children [24]. Irisin was recognized as an independent negative predictor of both circulating CD144(+) and CD31(+)/CD42b(-) EMPs in the total study population, as well as of circulating CD31(+)/CD42b(-) EMPs in the preterm-born group, after adjusting for age, sex, BMI, WHR, HOMA-IR and lipid levels. These findings are in line with the proposed protective role of irisin for the endothelium [24,30,31,32]. Irisin may be associated with reduced apoptosis of endothelial cells, since both CD144(+) and CD31(+)/CD42b(-) EMPs are biomarkers of endothelial apoptosis [49]. The decreased irisin levels along with the increased circulating EMPs in the preterm-born group are indicative of endothelial dysfunction and endothelial cell apoptosis in this group.

The decreased irisin levels in our preterm-born population are consistent with the results of previous studies in preterm and SGA neonates, as well as in children born prematurely [33,34,35]. The suppression of irisin production in preterm and SGA neonates and children might be due to their reduced muscular mass and brown adipose tissue [34,35,50]. Moreover, adipose tissue partitioning alterations and, specifically, the prematurity-related predisposition to a more central fat deposition pattern may be responsible for the decreased irisin levels in the preterm-born individuals compared to full-term peers [33,51] since visceral adipose tissue has a minor contribution to the production and signaling of FNDC5/irisin in comparison with subcutaneous adipocytes [51].

In our study population, plasma irisin was also an independent predictor of both CD34(+)/VEGFR-2(+)/CD45(-) and CD34(+)/VEGFR-2(+)/CD45dim EPCs. However, contrary to expectations [30,31], the association between irisin and EPCs concentrations was found to be inverse; the lower the irisin, the higher the EPCs. As children are not “just small adults”, we suggest that EPCs can increase in children in order to counteract the endothelial dysfunction and damage. This hypothesis can explain the increased circulating EPCs despite the increased EMPs and decreased irisin levels in preterm-born children as compared to full-term ones.

The strength of our study includes the novelty of the results, as well as the investigation of irisin correlations with significant EMP and EPC subpopulations in prepubertal preterm-born children compared to full-term peers. In this study, we aimed on three different EMP subpopulations -CD62E(+), CD144(+) and CD31(+)/CD42b(-) EMPs- using surface markers expressed only on endothelial cells and considered as the most endothelium-specific [7,8,10,49]. Regarding the cell surface markers used to detect EPCs, the dual expression of an immaturity/stem antigen (CD34 and/or CD133) and of an endothelial antigen (VEGFR-2) was implemented, as previously described; the CD45(-), and especially the CD45dim population, provide more precision to the identification of EPCs [52,53,54]. Furthermore, associations of irisin with several anthropometric and metabolic parameters were also examined. All the above are the main strengths of this study.

This study has some limitations. Firstly, although a sufficient number of preterm-born children was included in order to obtain significant results for the primary outcome measures, this number was not adequate enough to reveal possible associations between plasma irisin levels and circulating EMPs and EPCs regarding preterm-birth-related maternal and/or neonatal morbidity, such as gestational hypertension and preeclampsia, BPD, IVH, ROP, etc. Including more neonatal units would have led to an increase in study participants and could further strengthen the results. Secondly, we identified EPCs by phenotyping without investigating their proliferative, migratory or clonogenic capacity. However, it is of great significance that both circulating CD34(+)/VEGFR-2(+)/CD45(-) and CD34(+)/VEGFR-2(+)/CD45dim EPCs are considered as the most specific EPCs subpopulations for their role in vascular integrity, endothelial remodeling and neovascularization [52,53,54]. 

## 5. Conclusions

In conclusion, plasma irisin levels are lower in prepubertal children born prematurely compared to controls, and correlate independently with circulating EMP and EPC subpopulations in the total study population and in the preterm-born group. However, further research on the association of irisin with circulating EMPs and EPCs in preterm-born individuals is required, including in vitro and experimental models, to better clarify and improve knowledge regarding the potential pathways underlying the link between irisin and the early stages of prematurity-related endothelial dysfunction.

## Figures and Tables

**Table 1:** Clinical, neonatal and perinatal characteristics, and biochemical profile of study participants.

Variable	Preterm-Born Children (*n* = 61)	Controls (*n* = 70)	*p* Value
Age (years)	10.9 ± 1.8	10.5 ± 1.9	0.43
Males (*n*)	24	36	0.17
Small for gestational age (SGA)	10	8	0.37
Gestational hypertension [*n* (%)]	2 (3.3)	0 (0)	N/A
Preeclampsia [*n* (%)]	8 (13.1)	1 (1.4)	**0.01**
Gestational diabetes [*n* (%)]	10 (16.4)	9 (12.9)	0.57
Cesarean delivery [*n* (%)]	57 (93.4)	34 (48.6)	**<0.001**
Gestational age (weeks)	31.7 ± 3.2	38.9 ± 1.0	**<0.001**
Birth weight (g)	1619 ± 540	3230 ± 457	**<0.001**
Bronchopulmonary dysplasia (BPD) [*n* (%)]	12 (19.7)	0 (0)	N/A
Intraventricular hemorrhage (IVH) [*n* (%)]	16 (26.2)	0 (0)	N/A
Retinopathy of prematurity (ROP) [*n* (%)]	22 (36.1)	0 (0)	N/A
Patent ductus arteriosus (PDA) [*n* (%)]	8 (13.1)	0 (0)	N/A
Physical activity rating (PA-R)	4 (3–7)	5 (3–7)	0.45
Body weight (kg)	41.6 ± 11.5	38.0 ± 9.6	0.11
Height (cm)	145.8 ± 11.9	142.9 ± 12.4	0.22
Body mass index (BMI) (kg/m^2^)	19.2 ± 3.3	18.3 ± 2.7	0.14
Waist circumference (cm)	71.9 ± 9.7	68.0 ± 8.3	**0.02**
Hip circumference (cm)	80.2 ± 10.0	77.2 ± 8.5	0.08
Waist-to-hip ratio (WHR)	0.90 ± 0.05	0.88 ± 0.04	**0.05**
Neck circumference (cm)	29.9 ± 2.5	29.0 ± 1.8	**0.05**
Systolic blood pressure (SBP) (mmHg)	107.5 ± 11.4	102.4 ± 9.1	**0.01**
Diastolic blood pressure (DBP) (mmHg)	66.6 ± 7.6	63.8 ± 7.0	**0.04**
Glucose (mg/dL)	78.6 ± 8.7	79.8 ± 7.0	0.43
Insulin (μUI/mL)	6.8 (5.1–9.8)	6.7 (5.7–8.8)	0.70
HOMA-IR	1.26 (0.94–1.95)	1.30 (1.07–1.67)	0.66
Total Cholesterol (mg/dL)	150.0 (134.5–170.0)	156.5 (141.0–166.5)	0.25
HDL-C (mg/dL)	63.5 ± 13.6	63.0 ± 14.2	0.79
LDL-C (mg/dL)	73.0 (60.0–93.5)	80.0 (66.3–93.8)	0.28
Triglycerides (mg/dL)	51.0 (40.0–68.0)	56.5 (41.0–72.3)	0.70

HOMA-IR, the homeostasis model assessment index; HDL-C, high-density lipoprotein cholesterol; LDL-C, low-density lipoprotein cholesterol; N/A, not applicable. Statistical significance is defined by *p* value ≤ 0.05. Significant results are shown in bold.

**Table 2:** Multiple regression analyses for independent associations of endothelial microparticles (EMPs) and endothelial progenitor cells (EPCs) with irisin levels, anthropometric and metabolic parameters in the total study population and in the preterm-born group.

Multiple Regression Analyses *			
Dependent Variables	Independent Variables with Significant Association	Standardized Coefficient Beta	*p*-Value
**Total study population**			
A. CD144(+) EMPs	Irisin (ng/mL)	−0.23	0.01
B. CD31(+)/CD42b(-) EMPs	Irisin (ng/mL)	−0.27	0.002
	Waist-to-hip ratio	−0.21	0.02
C. CD34(+)/VEGFR-2(+)/CD45(-) EPCs	Irisin (ng/mL)	−0.25	0.003
	Age (years)	0.19	0.02
	Triglycerides (mg/dL)	−0.25	0.003
D. CD34(+)/VEGFR-2(+)/CD45dim EPCs	Irisin (ng/mL)	−0.34	<0.001
	Age (years)	0.19	0.02
	Triglycerides (mg/dL)	−0.30	<0.001
**Preterm-born group**			
E. CD31(+)/CD42b(-) EMPs	Irisin (ng/mL)	−0.32	0.01
	SGA	0.25	0.03

* Independent variables in all models: irisin, age, sex, born SGA, BMI, Waist-to-hip ratio, HOMA-IR, total cholesterol and triglyceride levels. SGA, small for gestational age; BMI, body mass index; HOMA-IR, the homeostasis model assessment index. Statistical significance is defined by *p* value ≤ 0.05.

## Data Availability

The data presented in this study are available on reasonable request and should be made to Tania Siahanidou (siahan@med.uoa.gr). The data are not publicly available due to privacy.

## Supplementary Materials

The following supporting information can be downloaded at: https://www.mdpi.com/article/10.3390/metabo13010120/s1, Figure S1: Circulating CD62E(+), CD144(+) and CD31(+)/CD42b(−) endothelial microparticles (EMPs) in preterm-born children compared to controls. Mean values of CD62E(+) (a), CD144(+) (b), and CD31(+)/42b(−) EMPs (c) in children born prematurely and controls. Error bars represent standard error of mean; Figure S2: Circulating CD34(+)/VEGFR-2(+)/CD45(-) (a) and CD34(+)/VEGFR-2(+)/CD45dim (b) endothelial progenitor cells (EPCs) in preterm-born children compared to controls. Mean values of CD34(+)/VEGFR-2(+)/CD45(-) and CD34(+)/VEGFR-2(+)/CD45dim EPCs in children born prematurely and controls. Error bars represent standard error of mean; Figure S3. Correlation of plasma irisin levels with CD62E(+) (r_s_ = −0.19, *p* = 0.03) (a), CD144(+) (r_s_ = −0.24, *p* = 0.01) (b) and CD31(+)/CD42b(-) (c) EMPs (r_s_ = −0.31, *p* < 0.001), as well as with CD34(+)/VEGFR-2(+)/CD45(-) (d) (r_s_ = −0.23, *p* = 0.01) and CD34(+)/VEGFR-2(+)/CD45dim (e) EPCs (r_s_ = −0.34, *p* < 0.001) in the total study population. EMPs and EPCs subpopulations were log-transformed before correlation analysis; Figure S4. Correlation of plasma irisin levels with CD62E(+) (a) (rs = −0.27, *p* = 0.03) and CD31(+)/CD42b(-) (b) EMPs (rs = −0.36, *p* = 0.004), as well as with CD34(+)/VEGFR-2(+)/CD45dim (c) EPCs (rs = −0.26, *p* = 0.04) in the preterm-born population. EMPs and EPCs subpopulations were log-transformed before correlation analysis.
