# Supplementary material for: Association of Plasma Irisin Levels with Circulating Endothelial Microparticles (EMPs) and Endothelial Progenitor Cells (EPCs) in Children Born Prematurely
Source: Metabolites. 2023 Jan 13;13(1):120. doi: 10.3390/metabo13010120 (PMC9861173; doi:10.3390/metabo13010120)
Supplement: Supplementary file 1 [file metabolites-13-00120-s001.zip › metabolites-2132368-supplementary.pdf]

Supplementary material

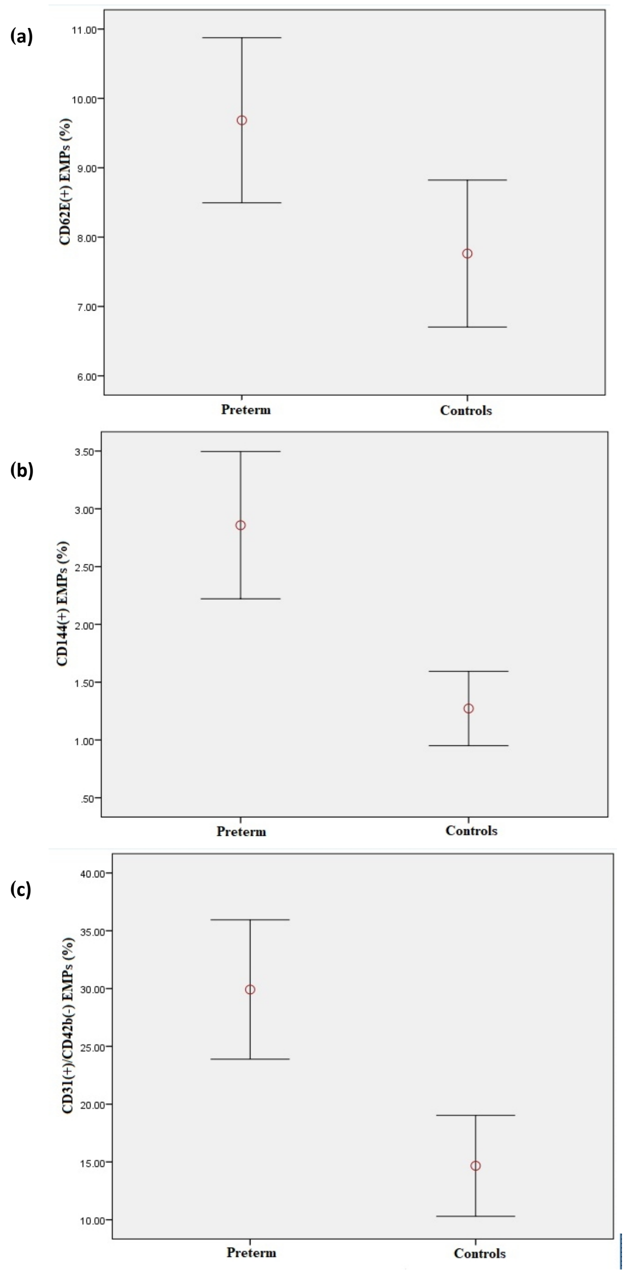

Figure S1. Circulating CD62E(+), CD144(+) and CD31(+)/CD42b(−) endothelial microparticles (EMPs) in preterm-born children compared to controls. Mean values of CD62E(+) (a), CD144(+) (b), and CD31(+)/42b(−) EMPs (c) in children born prematurely and controls. Error bars represent standard error of mean.

(a)

(b)

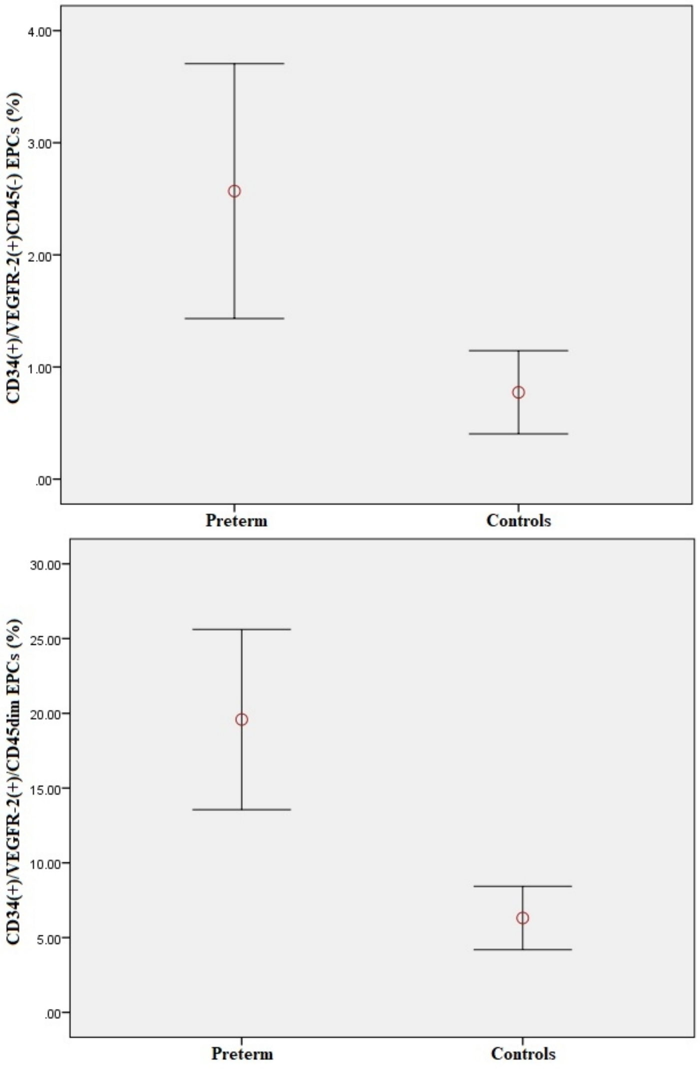

Figure S2. Circulating CD34(+)/VEGFR-2(+)/CD45(-) (a) and CD34(+)/VEGFR-2(+)/CD45dim (b) endothelial progenitor cells (EPCs) in preterm-born children compared to controls. Mean values of CD34(+)/VEGFR-2(+)/CD45(-) and CD34(+)/VEGFR-2(+)/CD45dim EPCs in children born prematurely and controls. Error bars represent standard error of mean.

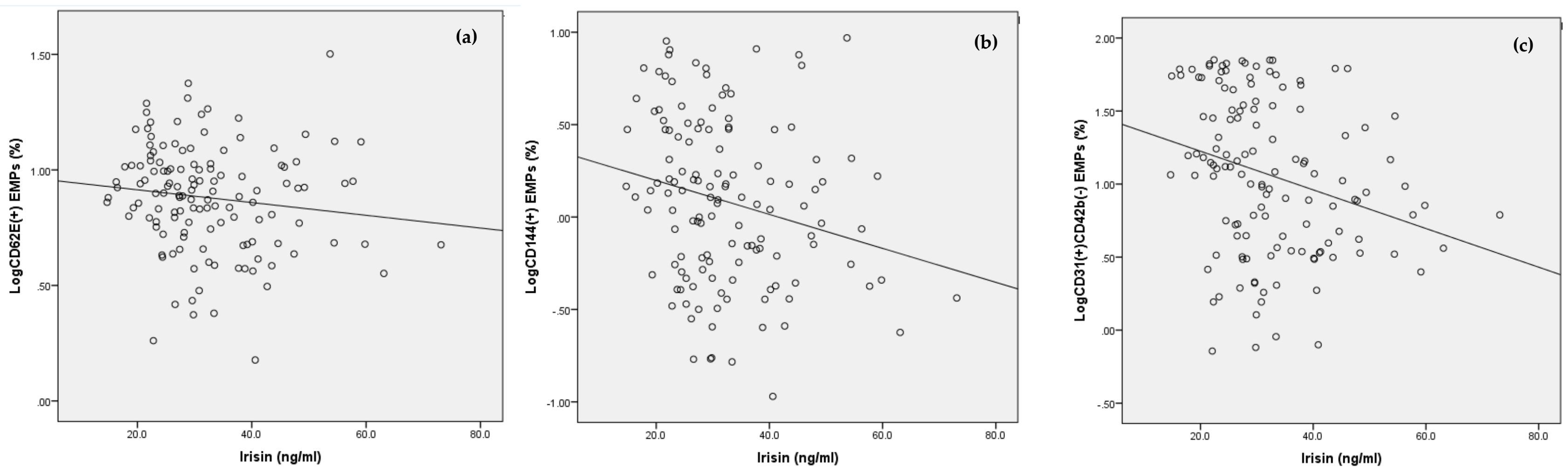

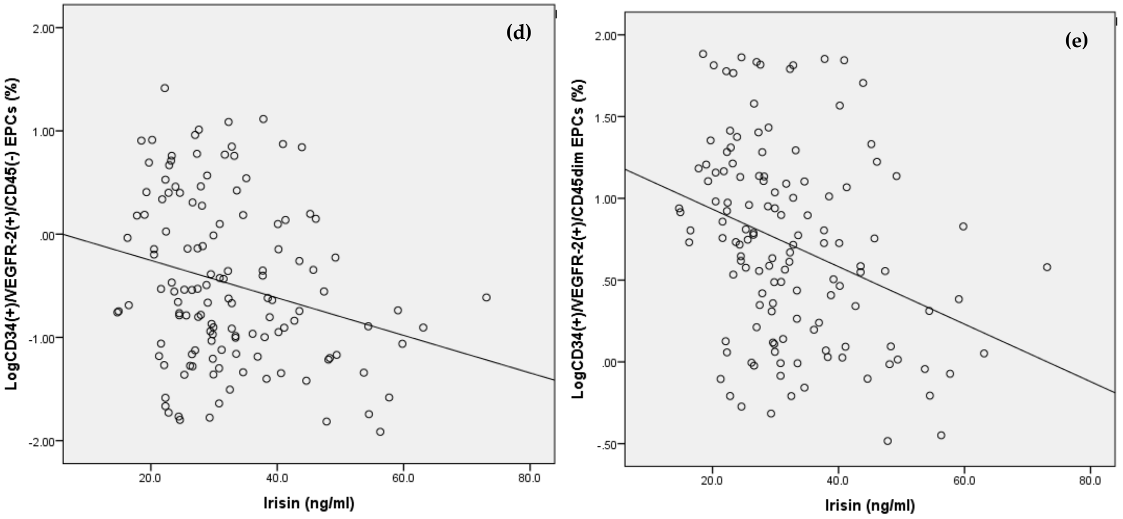

Figure S3. Correlation of plasma irisin levels with CD62E(+) (r_s=-0.19, p=0.03) (a), CD144(+) (r_s=-0.24, p=0.01) (b) and CD31(+)/CD42b(-) (c) EMPs (r_s=-0.31, p<0.001), as well as with CD34(+)/VEGFR-2(+)/CD45(-) (d) (r_s=-0.23, p=0.01) and CD34(+)/VEGFR-2(+)/CD45dim (e) EPCs (r_s=-0.34, p<0.001) in the total study population. EMPs and EPCs subpopulations were log-transformed before correlation analysis.

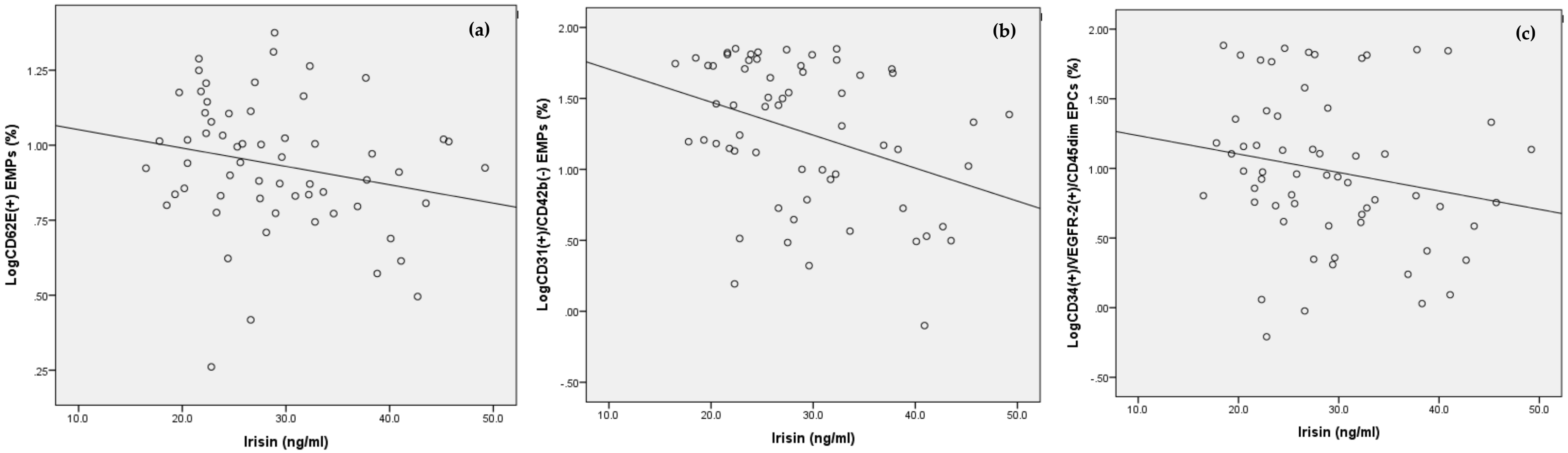

Figure S4. Correlation of plasma irisin levels with CD62E(+) (a) (r_s=-0.27, p=0.03) and CD31(+)/CD42b(-) (b) EMPs (r_s=-0.36, p=0.004), as well as with CD34(+)/VEGFR-2(+)/CD45dim (c) EPCs (r_s=-0.26, p=0.04) in the preterm-born population. EMPs and EPCs subpopulations were log-transformed before correlation analysis.
